# Dynamics of Inflammatory Factors in Aqueous Humor During Brolucizumab Treatment for Age-Related Macular Degenerations: A Case Series

**DOI:** 10.3390/medicina61030372

**Published:** 2025-02-20

**Authors:** Masaki Asakage, Hidetaka Noma, Kanako Yasuda, Hiroshi Goto, Masahiko Shimura

**Affiliations:** 1 Department of Ophthalmology, Hachioji Medical Center, Tokyo Medical University, 1163, Tatemachi, Hachioji, Tokyo 193-0998, Japan; patty.m.best@gmail.com (M.A.); kana6723@yahoo.co.jp (K.Y.); masahiko@v101.vaio.ne.jp (M.S.); 2 Department of Ophthalmology, Tokyo Medical University, Tokyo 160-8402, Japan; goto1115@tokyo-med.ac.jp

**Keywords:** age-related macular degeneration, brolucizumab, intraocular inflammation, monocyte chemoattractant protein 1, interleukin-6, interleukin-8, interferon-inducible 10 kDa protein, Fms related tyrosine kinase 3 ligands, fractalkine

## Abstract

Anti-vascular endothelial growth factor (VEGF) treatment with intravitreal brolucizumab (IVBr) was launched as a novel treatment for neovascular age-related macular degeneration (AMD), but the incidence of intraocular inflammation (IOI) as a specific adverse effect of brolucizumab has been reported. We evaluated the dynamics of inflammatory factors in AMD in patients with or without IOI before and after anti-VEGF treatment with IVBr. We describe three patients who did not develop inflammation after three consecutive administrations of IVBr and three in whom inflammation occurred after the first IVBr treatment. The presence or absence of inflammation was determined by slit-lamp examination and a laser flare meter. Aqueous humor was obtained during anti-VEGF treatment with IVBr. Levels of VEGF, platelet-derived growth factor (PDGF)-AA, monocyte chemoattractant protein 1 (MCP-1), interleukin (IL)-6, IL-8, interferon-inducible 10 kDa protein (IP-10), Fms-related tyrosine kinase 3 ligands (Flt-3L), and fractalkine were measured. Vision worsened in one patient who developed IOI after initial IVBr, so IVBr was discontinued and the patient was switched to intravitreal aflibercept with sub-tenon injection of triamcinolone acetonide. IVBr was continued in the two other patients with IOI. VEGF decreased after IVBr in all patients with and without IOI. On the other hand, at 1 month IL-6, IL-8, MCP-1, IP-10, and Flt-3L were higher in the three patients with IOI compared with baseline and with the three patients without IOI. In two patients with IOI, not only flares but also IL-8, IP-10, and Flt-3L decreased from 1 to 2 months after IVBr despite continued IVBr. This case series might lead to a better understanding of the pathogenesis of IOI after IVBr.

## 1. Introduction

Anti-vascular endothelial growth factor (VEGF) treatment with the monoclonal antibody brolucizumab (Beovu, Novartis) was launched in the USA in October 2019 as a novel treatment for neovascular age-related macular degeneration (AMD) [1,2].

Brolucizumab has a low molecular weight, is administered at a high concentration, and easily penetrates the retina [1,2].

In terms of intraretinal fluid and subretinal fluid absorption (fluid control), brolucizumab has been reported to have superior efficacy to aflibercept (Eylea; Regeneron and Bayer HealthCare) [1,2]. Several real-world studies on brolucizumab demonstrated favorable visual and anatomical treatment outcomes in AMD [3,4]. Additionally, brolucizumab showed a robust anatomical response in cases resistant to other anti-VEGF treatments, such as ranibizumab and aflibercept, indicating the potential of brolucizumab as an effective alternative treatment for refractory AMD [5,6]. However, the incidence of intraocular inflammation (IOI) as a specific adverse effect of brolucizumab has been reported to be 11% to 22% [4,7,8,9]. The dynamics of inflammatory factors before and after treatment with ranibizumab (Lucentis; Genentech) and aflibercept in AMD have been reported, but there are few reports on brolucizumab. Therefore, we present a case series that illustrates the changes in inflammatory factors in patients with and without IOI after treatment with brolucizumab.

## 2. Materials and Methods

We examined six treatment-naïve patients with neovascular AMD who were treated by intravitreal brolucizumab (IVBr) at the Department of Ophthalmology, Hachioji Medical Center, Tokyo Medical University, Tokyo, Japan. All patients provided written informed consent to treatment by IVBr, and treatment was approved by the institutional review board of Tokyo Medical University Hachioji Medical Center (IRB No. T2023-0039). All patients were 45 years of age or older, and none had myopia of more than -6 diopters; a history of uveitis or vitrectomy; type 2 diabetes or diabetic retinopathy; advanced cataracts or other choroidal or retinal pathologies; or previous ocular treatment with laser photocoagulation, intravitreal injection of another anti-VEGF agents, or surgery. A thorough ophthalmologic examination was performed, including best-corrected visual acuity (BCVA), central macular thickness (CMT), and fundus examinations, and aqueous flare was assessed using a laser flare meter. Fluorescein angiography (FA) and indocyanine green angiography (ICGA) with spectral-domain optical coherence tomography (Heidelberg Engineering, Heidelberg, Germany) were performed at baseline. AMD was classified into macular neovascularization type 1 (occult type), type 2 (classic type), type 3 (retinal angiomatous proliferation), and polypoidal choroidal vasculopathy. Polypoidal choroidal vasculopathy was diagnosed if ICGA showed polypoidal lesions. BCVA was converted to logarithm of the minimal angle of resolution (logMAR) units. CMT was measured manually from the superior border of the retinal pigment epithelium to the inner retinal layer border at the foveal center.

In three patients, three IVBr treatments could be performed as an induction phase without inflammation (patients without IOI), and in the other three, inflammation developed occurred after the first IVBr (patients with IOI). The presence or absence of inflammation was determined by slit-lamp examination and a laser flare meter (FC-600, Kowa Co., Ltd., Tokyo, Japan); i.e., in the slit-lamp examination, inflammation was defined as the presence of inflammatory cells in the anterior chamber, and in the laser flare meter examination, inflammation was defined as an aqueous flare value more than twice that of the pre-dose flare value.

For IVBr, topical anesthesia was administered and a 30 G needle was then used to obtain aqueous humor (mean, 0.1 mL); samples were stored in sterile plastic tubes at −80 °C until analysis. Then, brolucizumab was injected into the vitreous with a 30 G needle. For the injection, the needle was inserted through the pars plana in the superior temporal quadrant, 3.5 mm from the limbus. After IVBr, patients were instructed to apply antibiotic drops to the affected eye for 3 days.

Levels of VEGF, platelet-derived growth factor (PDGF)-AA, monocyte chemoattractant protein 1 (MCP-1), interleukin (IL)-6, IL-8, interferon-inducible 10 kDa protein (IP-10), Fms-related tyrosine kinase 3 ligands (Flt-3L), and fractalkine were measured in the aqueous humor samples by suspension array (xMAP; Luminex Corp., Austin, TX, USA) with the Milliplex kit (EMD Millipore, Burlington, MA, USA) [10,11,12,13,14,15]. A Human Cytokine/Chemokine kit (HCYTO-60K) was used for VEGF, PDGF-AA, MCP-1, IL-6, IL-8, IP-10, Flt-3L, and fractalkine. In accordance with the manufacturer’s instructions, fluorescence-labeled beads were mixed with standards and aqueous humor samples and incubated at 4 °C overnight. The next day, detection antibodies were added at room temperature for 1 h, followed by streptavidin-phycoerythrin at room temperature for 30 min. Readings were made with a Luminex 100/200 System (v5.1), and median fluorescent intensity was calculated with Milliplex Analyst software (v4.3). Concentrations were calculated by a 5-parameter logistic approach. All factors were present at detectable levels (minimum detectable concentrations: VEGF, 0.64 pg/mL; PDGF-AA, 0.64 pg/mL; MCP-1, 1.2 pg/mL; IL-6, 0.29 pg/mL; IL-8, 0.14 pg/mL; IP-10, 0.55 pg/mL; Flt-3L, 5.4 pg/mL; and fractalkine, 22.7 pg/mL).

We compared continuous variables with a paired *t* test and examined relationships among the variables by Pearson’s correlation analysis and linear regression analysis. Statistical significance was set as a *p* value of less than 0.05.

## 3. Results

The sociodemographic and clinical characteristics, BCVA, and CMT of the patients with and without IOI are presented in Table 1. VA fluctuation (logMAR) over the study period was 1.28, 0.18, and −0.08 in the IOI group and −0.20, 0, and −0.18 in the non-IOI group.

**Figure 1:**
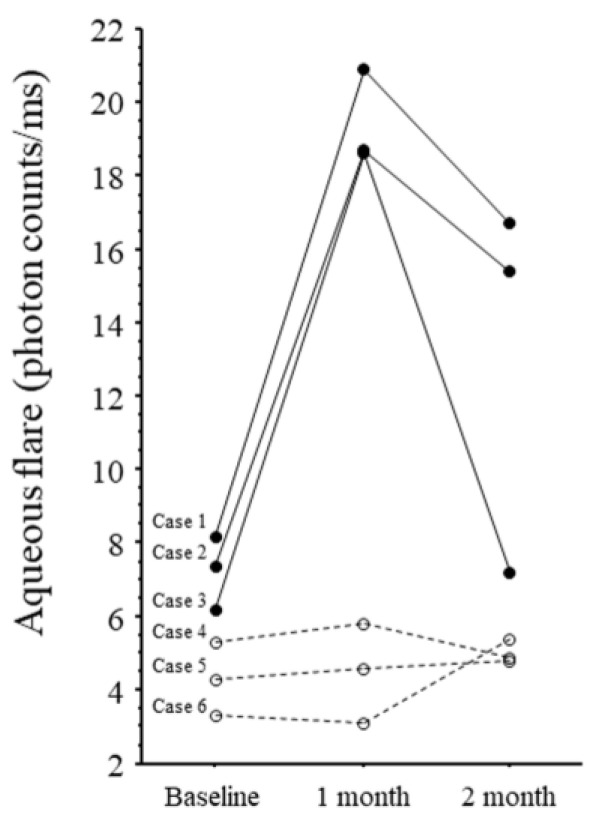
Changes in aqueous flare values in six patients. Cases 1–3: Patients with intraocular inflammation (solid line). Cases 4–6: Patients with non-intraocular inflammation (dotted line).

Cases 1 to 3 had IOI, and cases 4 to 6 did not. IVBr was discontinued in one patient (case 1) in whom vision worsened after the initial IVBr and IOI was confirmed; the patient was switched to intravitreal aflibercept and a sub-tenon injection of triamcinolone acetonide. In the other two cases of IOI, IVBr was continued because vision was not affected by the inflammation. The changes in the aqueous flare values are shown in Figure 1.

The aqueous flare value was highly elevated in all three IOI patients at 1 month compared with baseline and with the non-IOI group, but it decreased from 1 to 2 months. In the two patients with IOI who continued treatment (cases 2 and 3), the aqueous flare value decreased from 1 to 2 months despite continued IVBr.

**Figure 2:**
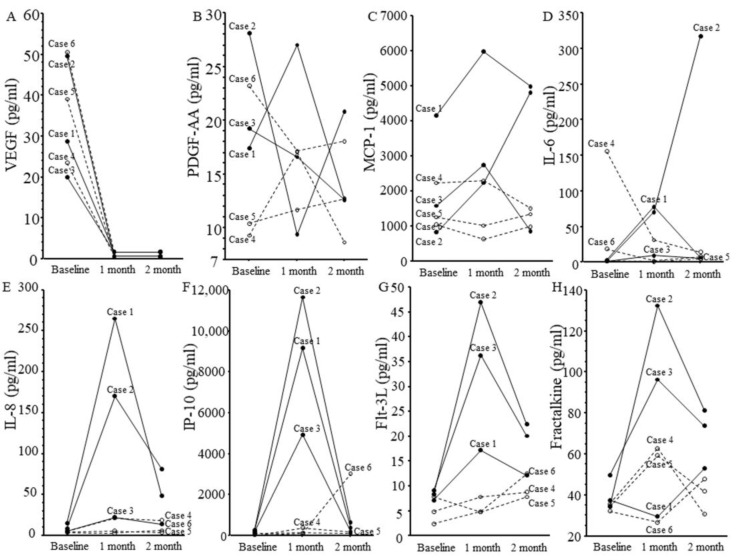
Changes in levels of eight cytokines in six patients. Cases 1–3: Patients with intraocular inflammation (solid line). Cases 4–6: Patients with non-intraocular inflammation (dotted line). (**A**) Vascular endothelial growth factor, (**B**) platelet-derived growth factor AA, (**C**) monocyte chemoattractant protein 1, (**D**) interleukin (IL)-6, (**E**) IL-8, (**F**) interferon-inducible 10 kDa protein, (**G**) Fms-related tyrosine kinase 3 ligand, and (**H**) fractalkine.

The dynamics of VEGF, PDGF-AA, MCP-1, IL-6, IL-8, IP-10, Flt-3L, and fractalkine are shown in Figure 2.

In all patients, VEGF had significantly decreased at 1 month after IVBr (*p* = 0.001). IL-6, IL-8, MCP-1, IP-10, and Flt-3L were highly elevated in all three patients with IOI at 1 month compared with baseline and with the non-IOI group, but IL-8, IP-10, and Flt-3L decreased from 1 to 2 months. In the two patients with IOI who continued treatment (cases 2 and 3), IL-8, IP-10, and Flt-3L decreased from 1 to 2 months despite continued IVBr.

We found significant correlations between the aqueous flare value and aqueous humor level of IL-8 (y = 3.04 + 0.38x; r = 0.85, *p* = 0.03) but not between the aqueous flare value and aqueous humor level of the other factors (i.e., VEGF, PDGF-AA, MCP-1, IL-6, IP-10, fractalkine, and flt-3 ligand).

## 4. Discussion

This case series indicates that VEGF decreases after IVBr in patients with non-IOI and IOI. Changes in VEGF after anti-VEGF treatment were consistent with previous reports [8,16,17,18]. This result was reasonable because IVBr potently suppresses VEGF. On the other hand, IL-6, IL-8, MCP-1, IP-10, and Flt-3L were highly elevated in all three patients with IOI at 1 month compared with baseline and with non-IOI patients, suggesting an inflammatory response in the eye. IOI and inflammation after IVBr are assumed to be due to hypersensitivity because the vitreous humor of IOI patients contains inflammatory cells involved in hypersensitivity reactions (CD4^+^ T helper cells, CD8^+^ T-cytotoxic cells, CD3^+^ T cells, CD20^+^ B cells, and CD68^+^ histiocytes) [16].

Because IOI developed about 1 month after IVBr, the reaction is assumed to be delayed type IV hypersensitivity [19,20]. This type of reaction is subdivided into types IVa (the classic hypersensitivity reaction), IVb, IVc, and IVd and is due to CD4^+^ T helper-1 (Th1) cell responses to antigens presented by antigen-presenting cells (APCs) [21]. In type IVa reactions, APCs secrete interferon gamma, tumor necrosis factor alpha, chemokine ligand 2 (CCL2), and IL-18 and then activate macrophages; in type IVb reactions, APCs sensitize CD4^+^ T-helper 2 (Th2) cells, leading to the production of IL-4, IL-5, and IL-13, the survival and migration of eosinophils, and, subsequently, inflammation; type IVc reactions involve the secretion by cytotoxic CD8^+^ T cells of B perforin, granulysin, and granzyme; and type IVd reactions involve the secretion by neutrophils of pro-inflammatory cytokines after activation by T cells [22,23]. Various pro-inflammatory cytokines play a role in these hypersensitivity reactions. Studies have shown that IL-6 levels increase in hypersensitivity reactions that occur after administration of monoclonal antibodies such as brolucizumab, indicating that IL-6 may be useful as a biomarker [24,25].

In this case series, MCP-1, IP-10, and Flt-3L levels were higher in all patients with IOI at 1 month compared with both baseline and the three non-IOI patients. As regards MCP-1, animal studies have shown that MCP-1 causes macrophages to migrate into choroidal neovascularization lesions in AMD and is involved in the digestion of both Bruch’s membrane and the retinal pigment epithelium [26,27]. Compared with mice treated with vehicle, mice treated with the angiotensin II type 1 receptor blocker telmisartan had less intraocular expression of MCP-1 mRNA, fewer retinal adherent leukocytes, weaker responses of lymph node cells to human interphotoreceptor retinoid binding protein-derived peptide 1–20, and significantly fewer CD44 high CD4^+^ T cells [28].

IP-10 is a member of the CXC family of chemokines and considered to play an important role in inflammation because of its T-cell chemotactic and adhesion-promoting properties [29,30]. Both a Th1-like (interferon gamma, IP-10, CXC motif chemokine receptor 3) and a Th2-like (IL-5, IL-4 and STAT-6) pathway contribute to eosinophil recruitment in early delayed-type hypersensitivity [31].

Flt-3L is a cytokine known to be involved in the mobilization of hematopoietic stem cells and their differentiation to cells such as conventional dendritic cells (cDCs), which play important roles in T-cell immune responses [32,33]. A congenital deficiency of cDCs leads to abnormalities in the immune system, such as changes in the composition and function of granulocytes, group 2 innate lymphoid cells, and B cells, which may be due to a breakdown of the Flt-3L-mediated homeostatic feedback loop [34]. The above findings suggest that IOI involves the inflammatory cytokines that drive type IV hypersensitivity and the immune responses described above.

In this study, all three patients with IOI had high flare values before treatment compared with non-IOI patients (Figure 1). This finding suggests that patients with high pre-treatment flare values = already have high levels of inflammation and, consequently, are more prone to IOI. If we used an aqueous flare cut-off value of 6, aqueous flare values were all above 6 in the IOI group, indicating that a baseline aqueous flare value above 6 may be associated with an IOI. VEGF induces production of nitric oxide, which causes blood vessels to dilate [35], so anti-VEGF therapy induces retinal arteriolar vasoconstriction [36,37,38]. Therefore, the elevated cytokine levels in the patients with IOI may be due not only to a delayed response, but also to the relative ischemia from vasoconstriction caused by IVBr because the ischemia may further increase inflammation and cause levels of these cytokines to rise. This hypothesis is supported by our previous finding that the aqueous flare value was significantly correlated with the size of the nonperfused area of the retina [39]. Taken together, findings suggest that patients with high pre-treatment flare values have a high inflammatory status and may be at risk of developing IOI, so care needs to be taken when administering IVBr in these patients. Further research on IVBr and IOI is needed.

Interestingly, in two of the three the patients with IOI (cases 2 and 3), not only aqueous flare values but also IL-8, IP-10, and Flt-3L levels decreased from 1 to 2 months despite continued IVBr. These changes suggest that inflammation was subsiding. IL-8 is secreted by cells with Toll-like receptors and is involved in the innate immune response [40]. IP-10 is a chemoattractant for macrophages, dendritic cells, and T cells, is involved in type 1 immune responses mediated by Th-1 and Th2 cells, and generally activates cell-based immunity [29,30]. Higher expression levels of Flt-3L have been linked to autoimmune and chronic inflammatory responses in the lung, central nervous system, and gastrointestinal tract [41]. These findings indicate that inflammation may have decreased because of the effects of various immune mechanisms. Further studies are needed to determine whether and, if so, why inflammation decreases over time in patients with IOI.

The innate immune system involves complex intrinsic interactions between cytokines in which cytokines interact to drive multicellular responses, including vascular and neuronal inflammation [42]. Previously, we reported significant correlations between aqueous flare value and aqueous humor levels of MCP-1, IL-6, IL-8, and IP-10 [14]. Recently, significant correlations were found among CCL2, CXC motif chemokine ligand 1, IL-6, IL-8, IL-10, granulocyte colony stimulating factor, granulocyte-macrophage colony-stimulating factor, intercellular adhesion molecule 1, E-selectin, and VEGF in IOI patients [43]. These results indicate that interactions among inflammatory cytokines are involved in the development of IOI. In addition, in this series, the IL-8 concentration was significantly correlated with the aqueous flare value, suggesting that IL-8 is a key factor in increasing intraocular inflammation after IVBr. However, the small number of patients means that further investigation is needed to clarify the relationship between IOI and inflammatory factors before and after IVBr in patients with AMD.

The study has some limitations, including the small sample size (*n* = 6), limited information on patient background, and short study period (1–2 months). Furthermore, in case 1, the fractalkine level decreased despite the presence of IOI after injection. The available data on this case do not explain why this change occurred, so the effects of IVBr on fractalkine require further study.

## 5. Conclusions

In conclusion, this case series might lead to a better understanding of the pathogenesis of IOI after IVBr.

## Figures and Tables

**Table 1:** Demographic characteristics.

	Case 1	Case 2	Case 3	Case 4	Case 5	Case 6
Age (years) Sex (Female/Male)	75 M	70 M	83 M	77 M	65 M	55 M
AMD type Intraocular inflammation	PCV +	PCV +	MNV1 +	PCV −	MNV1 −	PCV −
BCVA (logMAR)						
Baseline 1 month 2 months	0.22 2.0 1.5	0.52 0.7 0.7	0.3 0.4 0.22	1.7 1.5 1.5	0 −0.08 0	0.10 0.05 −0.08
CMT (μm) Baseline 1 month 2 months	680 809 555	389 211 184	267 208 202	171 152 131	362 201 205	390 230 195

AMD = age-related macular degeneration; BCVA = best-corrected visual acuity; CMT = central macular thickness; logMAR = logarithm of the minimal angle of resolution; PCV = polypoidal choroidal vasculopathy; MNV = macular neovascularization.

## Data Availability

The datasets used and/or analyzed during the current study are included in this article.

## Abbreviations

The following abbreviations are used in this manuscript:

AMD	age-related macular degeneration
BCVA	best-corrected visual acuity
CMT	central macular thickness
FA	fluorescein angiography
Flt-3L	Fms-related tyrosine kinase 3 ligand
ICGA	indocyanine green angiography
IL	interleukin
IOI	intraocular inflammation
IP-10	interferon-inducible 10 kDa protein
IVBr	intravitreal brolucizumab
LogMAR	logarithm of the minimum angle of resolution
MCP-1	monocyte chemoattractant protein 1
MNV	macular neovascularization
OCT	optical coherence tomography
PCV	polypoidal choroidal vasculopathy
PDGF	platelet-derived growth factor
SD-OCT	spectral-domain OCT
SRD	serous retinal detachment
